# Identification, Characterization, and Mutational Analysis of a Probable KEAP1 Ortholog in Rice (*Oryza sativa* L.)

**DOI:** 10.3390/plants9111450

**Published:** 2020-10-27

**Authors:** Yan-Hua Liu, Meng Jiang, Rui-Qing Li, Rasbin Basnet, Jian-Zhong Huang, Shi-Yong Song, Qing-Yao Shu

**Affiliations:** 1 National Key Laboratory of Rice Biology and Zhejiang Key Laboratory of Crop Germplasm Resources, Institute of Crop Sciences, Zhejiang University, Hangzhou 310058, China; 11616004@zju.edu.cn (Y.-H.L.); mengjiang@zju.edu.cn (M.J.); 11416095@zju.edu.cn (R.B.); jzhuang@zju.edu.cn (J.-Z.H.); shiyongsong@zju.edu.cn (S.-Y.S.); 2 College of Agronomy, Anhui Agricultural University, Hefei 230036, China; 2018065@ahau.edu.cn; 3 Key Laboratory for Nuclear Agricultural Sciences of Zhejiang Province and Ministry of Agriculture and Rural Affairs, Institute of Nuclear Agricultural Sciences, Zhejiang University, Zijingang Campus, Hangzhou 310058, China

**Keywords:** redox, seed germination, abscisic acid (ABA), OsKEAP1, *Os01g0162500*, *Os05g0164900*, *Oryza sativa* L., CRISPR

## Abstract

The Kelch-like ECH-associated protein 1 (KEAP1)-nuclear factor E2-related factor 2 (NRF2) module is a key component in the detoxification and antioxidant system in animals, which plays crucial roles in cell homeostasis and cytoprotection, and consequently in carcinogenesis and disease development. However, this system seems to have diverged throughout evolution across different organisms, and the question of whether a similar system exists in plants has thus far remained unresolved. In this study, a KEAP1 ortholog was identified in rice (*Oryza sativa* L., OsKEAP1) and its properties were characterized via in silico and laboratory studies. To reveal OsKEAP1’s function, two knockdown mutants, *oskeap1-1* and *oskeap1-2*, were generated by targeted mutagenesis in the 5′ untranslated region (UTR) using the CRISPR-Cas9 system. In silico analysis showed that OsKEAP1 has a Kelch-repeat domain which is identical to those of animals and a plant-specific development and cell death (DCD) domain in place of the broad-complex, tramtrack, bric-a-brac (BTB) domain found in animals. Orthologs of OsKEAP1 are present across plant species and all have the DCD domain and the Kelch-repeat domain. OsKEAP1 was proven to be localized to both the cytoplasm and nucleus, in contrast to the exclusive cytoplasm localization of animal KEAP1. Single nucleotide insertions in the 5′ UTR significantly reduced the transcription level of *OsKEAP1* in the *oskeap1-1* and *oskeap1-2* mutants. The *oskeap1* mutations greatly impaired plant growth and development, resulting in significant declines in a majority of agronomic and yield-related traits, i.e., plant height, panicle length, grain number per plant, and seed-set rate. The downregulation of *OsKEAP1* increased the levels of H_2_O_2_, malondialdehyde, and proline while significantly decreasing the expression of two catalase genes in seedlings grown under normal and salt-stressed conditions. The changes in the above phenotypes are either positively or negatively correlated with the degree of OsKEAP1 downregulation. Altogether, we identified a probable KEAP1 ortholog in rice, revealed its unique subcellular localization, and demonstrated its important functions in vegetative and reproductive growth via regulation of the antioxidant response in plants.

## 1. Introduction

Cells have developed a myriad of antioxidant mechanisms conferring defense against various redox stresses. The KEAP1-NRF2 module has been recognized as one of the prominent components in defense systems involving antioxidant response in animals [1,2,3,4]. KEAP1 (Kelch-like ECH-associated protein 1) is a negative regulator of NRF2 (NF-E2 related factor) and is responsible for ubiquitination regulation of NRF2 in response to oxidative and electrophilic stress [5,6]. NRF2 is constantly ubiquitinated by the complex between KEAP1 and Cullin3-based ubiquitin E3 ligase, thereby inhibiting NRF2 activity in normal conditions [6]. NRF2 has been identified as a potent transcription regulator of many cytoprotective genes that defend cells, tissues, and organisms against various xenobiotics and oxidative stress, including inflammatory diseases of the lung, liver, and kidney, as well as neurodegenerative diseases such as Alzheimer’s and Parkinson’s diseases [7,8,9].

The KEAP1-NRF2 pathway has also been found in other organisms from arthropods to mammals, although the evidence as to whether a similar KEAP1-NRF2 system exists in plants and other organisms remains either contradictory or elusive. Through bioinformatics-based evolutional analyses, Gacesa R. et al. [10] concluded that the KEAP1-NRF2 system exists in organisms from arthropods to mammals, but not in early Eukarya, fungi, Nematoda, and basal metazoa. However, the same group did identify fungal KEAP1 and NRF2 orthologs, although they were distant and quite diverged from animal ones [11]. This discrepancy could be explained by the different parameters and methods used for the homolog search. However, Gacesa R et al. [11] did not identify any KEAP1 and NRF2 orthologs in plants.

Plants are sessile—their development, growth, and survival are continuously challenged by internal and external stresses. Plants have evolved several mechanisms that enable them to use their metabolism by integrating primary metabolic products into vital processes. Reactive oxygen species (ROS) are one such example of metabolic products that play an important role in the regulation of plant growth and development [12]. It is known that ROS levels are determined by a tightly controlled balance between production and breakdown that is achieved via fine-tuning of complex antioxidant systems [13]. Because no close KEAP1 and NRF2 orthologs have been identified in plants via a classical homolog search [11], there have been no experimental studies on candidate genes in plants. Consequently, the KEAP1-NRF2 system, though widely existing in animals and other organisms for redox regulation, has never even been discussed in plants. A brief search for KEAP1 orthologs in the rice genome, however, revealed that there are hypothetical putative proteins with the Kelch-repeat domain. This initial finding inspired the authors to explore the potential KEAP1-NRF2 module in plants using rice as a model.

In the present study, we first identified a rice KEAP1 ortholog (OsKEAP1) and characterized its properties, such as conserved domains and subcellular localization. We then developed two downregulated *OsKEAP1* mutants by means of targeted mutagenesis in its untranslated regions (UTRs) and investigated their agronomic and yield-related traits. Furthermore, we assessed the redox status and expression of a few genes known to be involved in redox under normal and abiotic-stressed conditions. Our results revealed that the downregulation of OsKEAP1 increased the level of H_2_O_2_, malondialdehyde, and proline and altered the expression of genes involved in the antioxidant response. Our present study thus reveals the function of OsKEAP1 in plant growth, development, and response to abiotic stresses and lays the basis for further investigation on whether there is a KEAP1-NRF2 module involved in ROS regulation in plants.

## 2. Results

### 2.1. Identification and Characterization of the KEAP1 Ortholog in Rice

**Figure 1:**
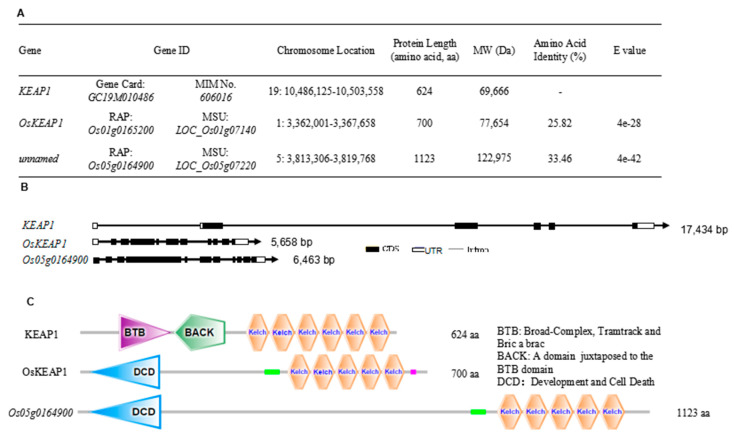
Gene information (**A**), structure (**B**), and protein features (**C**) of human KEAP1 and its orthologs in rice. Prediction of domains was performed at https://smart.embl.de/. The green and pink/red boxes represent the coiled-coil region and low complex region, respectively.

Two KEAP1-like genes were identified in the rice genome (c.v. Nipponbare) with relatively high coverages: *Os01g0162500* (54%) and *Os05g0164900* (46%). In the NCBI database, the former is annotated to encode a hypothetical Kelch-type beta-propeller domain-containing protein, and the latter a Kelch repeat type 1 domain-containing protein, hence both have the Kelch domain, as found in KEAP1. However, the amino acid identity score of the rice proteins to KEAP1 was only 25.82% and 33.46%, respectively (Figure 1A). Compared with *KEAP1*, both rice orthologs are much shorter in length but had more exons and introns. Both KEAP1 and its rice orthologs had relatively long untranslated regions (UTRs) at their 3′ termini (Figure 1B). The rice KEAP1 orthologs had five Kelch repeats, whereas KEAP1 had six Kelch repeats. At the N terminus, KEAP1 had a broad-complex, tramtrack, bric-a-brac (BTB-BACK) domain, whereas the rice orthologs had a development and programmed cell death (DCD) domain (Figure 1C). These characteristics suggest that KEAP1 and its rice orthologs might exhibit a similar basic function but respond to different development and environmental stimuli.

Because *Os01g0162500* encodes a protein of 700 amino acids (aa), similar to KEAP1 (624 aa), while *Os05g0164900* encodes a protein of 1123 aa, the peptide fragment between the DCD and Kelch domain of Os01g0162500 is far shorter than that of Os05g0164900. Hence, Os01g0162500 resembles KEAP1 more closely than Os05g0164900 does (Figure 1C). Therefore, *Os01g0162500* was closer to *KEAP1* than *Os05g0164900* and we, therefore, deemed it to be the most probable *KEAP1* ortholog in rice. Hereafter it is designated as *OsKEAP1* and studied further.

In silico analysis showed that *OsKEAP1* is mainly expressed in the inflorescence, anther, pistil, ovary, and embryo tissues (Figure S1). A search for proteins/peptides homologous to OsKEAP1/Os05g0164900 revealed 110 non-repeated sequences in plant species (Figure S2). Further analysis of a selection of KEAP1 orthologs in 10 crop plants showed that they are highly conserved in terms of protein length and domains, all having a DCD domain and 5–6 Kelch repeats (Figure S3).

**Figure 2:**
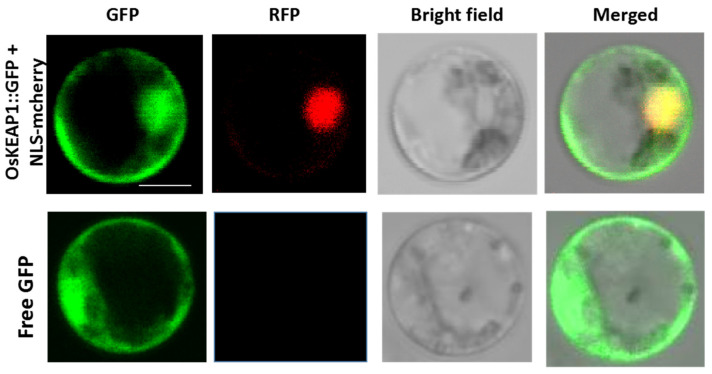
Transient expression of 35S:OsKEAP1:GFP fusion in rice protoplast. Protoplasts transfected with control vector (35S:GFP) have a bright GFP signal distributed throughout the cell, whereas those with 35S:OsKEAP1:GFP have a fluorescent signal (in green) localized in the nucleus and cytoplasm, confirmed by nuclear localization sequence (NLS) signal in the nucleus (in red). Bar = 5 µm.

To detect the subcellular localization of OsKEAP1, protoplasts transiently transformed with the *OsKEAP1::GFP* vector were observed under a fluorescence microscope. The results showed that the GFP signal from the control vector (pCAMBIA 1301) was found to be distributed throughout the cell, whereas the green fluorescent signal from GFP-fused proteins was transiently expressed in the nucleus and cytoplasm. The red fluorescence of NLS-mCherry overlapped with the green fluorescence, confirming the localization of OsKEAP1 in the nucleus, along with the cytoplasm (Figure 2).

### 2.2. Targeted Mutagenesis of 5′ UTR Downregulated OsKEAP1 Expression

**Figure 3:**
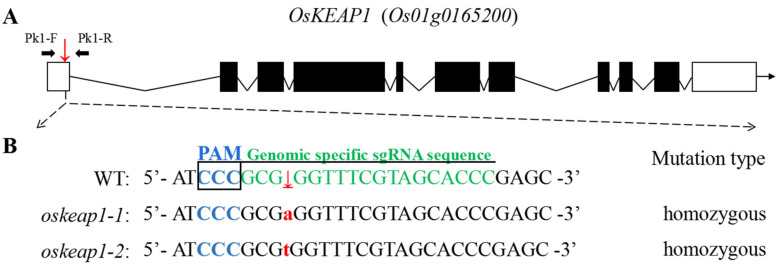
Targeted mutagenesis of *OsKEAP1* using the CRISPR/Cas9 system. (**A**) Gene structure of *OsKEAP1* with the sgRNA target shown by a frame; primers used for amplification of the fragment encompassing the target region (Pk1-F/R) are shown. (**B**) Sequence information around the target for the wild-type cultivar Xidao #1 (wild-type (WT)) and its two *oskeap1* mutants, the target sequence, and its PAM are highlighted.

In our early trials, we failed to generate knockout *oskeap1* mutants by targeting its first exon. The Biogle Gene Research team, who produced a large mutant library by CRISPR/Cas9-based targeted mutagenesis of virtually all rice genes [14], also failed to identify mutants when targeting two sites in the 4th exon and one site in the 9th exon. Therefore, we opted to produce knockdown mutants by targeting the UTR (Figure 3A). Among the T_0_ plants, two homozygous 1-bp insertion mutants were identified, one with an A (*oskeap1-1*) and the other with a T (*oskeap1-2*) in *OsKEAP1* (Figure 3B). The two plants were later developed into two transgene-free, stable mutant lines, which were named *oskeap1-1* and *oskeap1-2*.

**Figure 4:**
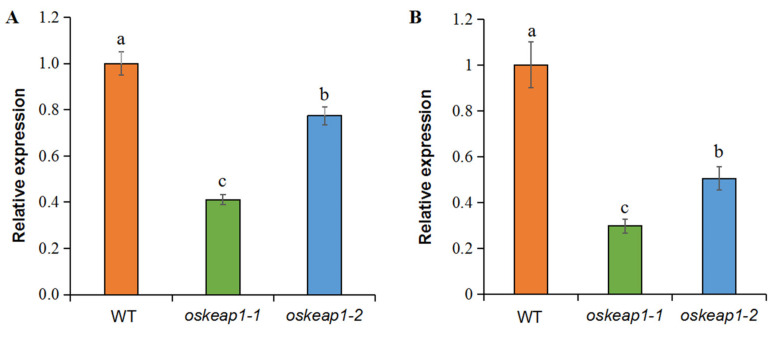
Relative expression of *OsKEAP1* in leaf tissues at seedling (**A**) and heading stage (**B**) of rice cultivar Xidao #1 (WT) and its two *oskeap1* mutants. The expression levels of the two mutants are compared with their wild-type at the same stage. *OsACTIN* was used as an internal reference. Data are shown in mean ± standard error of 3 biological repeats. Different letters (a,b,c) represent significant difference at the 0.05 level.

To assess whether and how the two insertion mutations affected the expression of *OsKEAP1*, qRT-PCR was performed to detect the abundance of *OsKEAP1* transcripts in the mutants and their wild-type parent Xidao #1. The results showed that, at both the seedling and flowering stage, the two mutants had significantly less abundant *OsKEAP1* transcripts than Xidao #1 (Figure 4). Comparatively, the mutational effect of *oskeap1-1* was more profound than that of *oskeap1-2*, with the abundance of *OsKEAP1* transcripts being only 41.09% (*oskeap1-1*) and 77.39% (*oskeap1-2*) that of Xidao #1 at the seedling stage (Figure 4A). The mutational effect became more significant at the flowering stage, with the *OsKEAP1* transcript level being only 29.77% and 50.46% that of the wild-type parent Xidao #1, respectively (Figure 4B).

### 2.3. Impact of OsKEAP1 Down-Regulation on Plant Growth and Development

**Figure 5:**
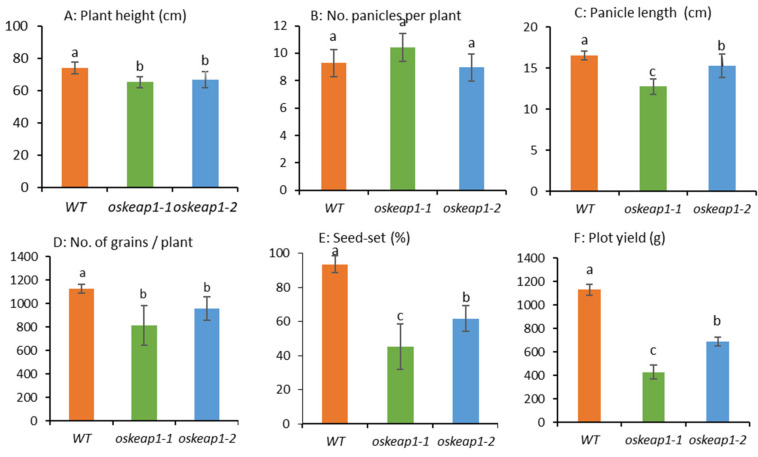
The agronomic and yield-related traits of rice wild-type cultivar Xidao #1 (WT) and its two *oskeap1* mutants. Values are mean ± SD of 3 replicates. Different letters represent significance at the 0.05 level.

The down-regulation of *OsKEAP1* had a significant negative effect on plant growth right from seed germination to seed maturing. The seven-day-old seedlings of both *oskeap1-1* and *oskeap1-2* were already obviously shorter than those of the wild-type (WT). At the maturity stage, compared with the WT, the two mutants had significantly shorter plant height than the WT, with a decrease of 11.8% and 10.0%, respectively (Figure 5A). Although the number of panicles per plant was not significantly different from that of the wild-type Xidao #1 (Figure 5B), the panicle length of the two mutants was significantly reduced, by 22.9% and 7.7%, respectively (Figure 5C).

**Figure 6:**
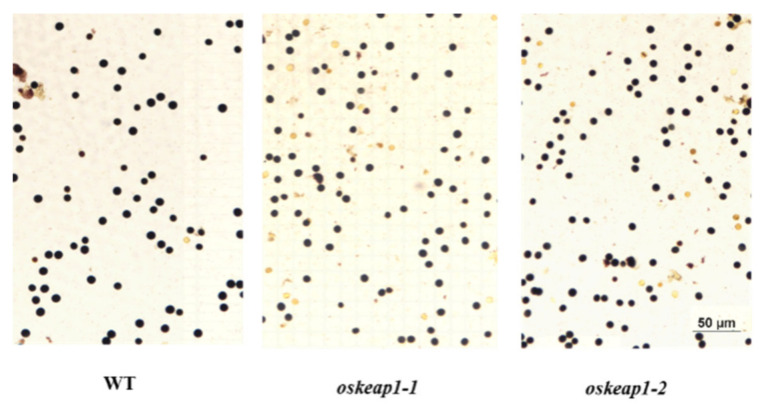
K-I_2_-stained rice pollen grains of Xidao #1 (WT) and its two *oskeap1* mutants.

The *oskeap1* mutations had profound effects on plant reproduction. The total grain number per plant was significantly reduced in *oskeap1-1* and *oskeap1-2*, by 27.5% and 14.9%, respectively (Figure 5D). More dramatic reductions were observed in the seed-set rate, with reductions of 52.0% and 31.8%, respectively, for *oskeap1-1* and *oskeap1-2* (Figure 5E). Consequently, the total seed yield per plot was significantly reduced in *oskeap1-1* and *oskeap1-2*, by 62.2% and 39.3%, respectively (Figure 5F). To examine whether the reduced seed-set rate was due to reduced male fertility, pollens were examined under a microscope after KI-I_2_ staining. The results showed that there were more unstained pollen grains in the two mutants than in wild-type parent Xidao #1 (Figure 6), i.e., the rate of stained pollen grains was 84.46%, 87.74%, and 96.14% in *oskeap1-1*, *oskeap1-2*, and wild-type Xidao #1, respectively.

### 2.4. Impact of OsKEAP1 Downregulation on Seed Germination and Sensitivity to Abscisic Acid (ABA)

**Figure 7:**
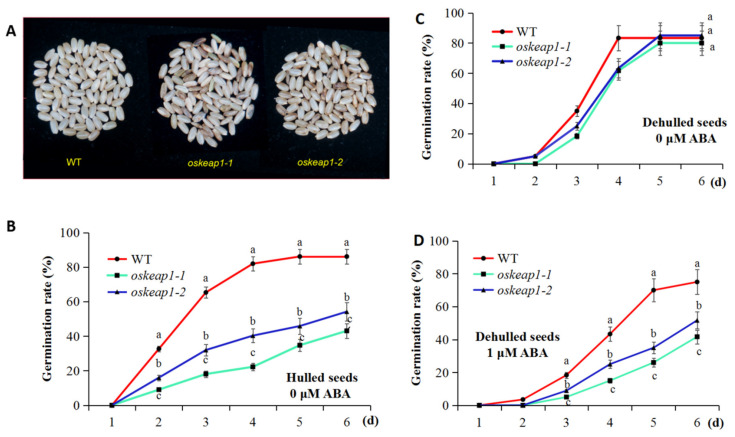
The phenotype of dehulled rice grains of wild-type Xidao#1 (WT) and its *oskeap1* mutants (**A**) and germination rate of hulled seeds (**B**) and dehulled seeds (brown rice) on medium without (**C**) or with (**D**) 1 μM abscisic acid (ABA). Data from the same observation date with different letters (a,b,c) are significantly different at the 0.05 level.

The *oskeap1* mutations also seemed to have an adverse effect on seed development, resulting in grains with black spots and wrinkles (Figure 7A). When tested using an intact rice grain for germination, *oskeap1-1* and *oskeap1-2* had a significantly lower seed germination rate, at 43.06% and 54.17% that of the wild-type parent Xidao #1 on day 6 (Figure 7B). To test whether the reduction of germination was related to seeds with black spots and wrinkles, rice grains were dehulled and brown rice grains without spots and wrinkles (normal looking grains) were selected for a germination test. No significant differences were observed between Xidao #1 and its two *oskeap1* mutants when grown on ½ MS medium [15] (Figure 7C), suggesting the grains with black spots/wrinkles contributed to the reduction of seed germination.

To further test whether OsKEAP1 is involved in the ABA signaling pathway, normal-looking mutant brown rice grains were tested for seed germination on ½ MS medium with 1 μM ABA. In this circumstance, *oskeap1* mutant seeds showed significantly lower germination rates, which suggests that they had become more sensitive to ABA than the wild-type Xidao #1 (Figure 7D).

### 2.5. Impact of OsKEAP1 Downregulation on Redox Regulation

**Figure 8:**
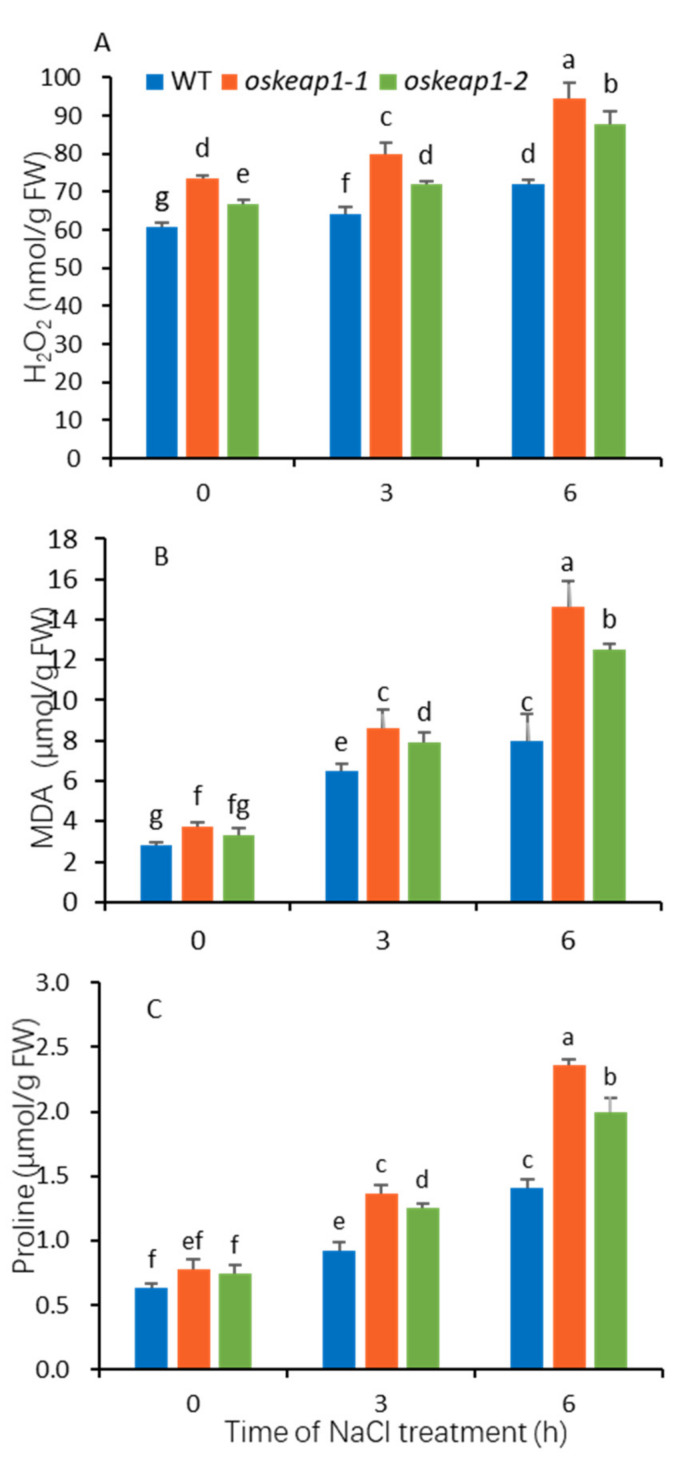
The content of H_2_O_2_, malondialdehyde (MDA), and proline in the above-ground tissues of Xidao #1 (WT) and its *oskeap1* mutants. Ten-day-old seedlings were treated with 200 mM NaCl for 3 and 6 h. Data with different letters (a,b,c, etc.) are significantly different at the 0.05 level.

To test the influence of *OsKEAP1* downregulation on redox status in rice, the H_2_O_2_ level was first measured in above-ground tissues of rice seedlings grown under normal and NaCl-stressed conditions. Under both normal and salt stress conditions, the two mutants had significantly higher H_2_O_2_ levels than the WT (Figure 8A). Salt treatment significantly increased the H_2_O_2_ level in both the WT and its two *oskeap1* mutants (Figure 8A).

Malondialdehyde (MDA) and proline are two indicators of redox status and plants often have increased levels of these two chemicals after being subjected to stress treatment. Under normal conditions, the two *oskeap1* mutants had MDA and proline levels similar to that of the WT, except that the MDA content of *oskeap1-1* was significantly different from that of the WT (Figure 8B). Salt treatment significantly increased the levels of MDA and proline in both the WT and its two *oskeap1* mutants, with the same trend as H_2_O_2_ (Figure 8B,C).

**Figure 9:**
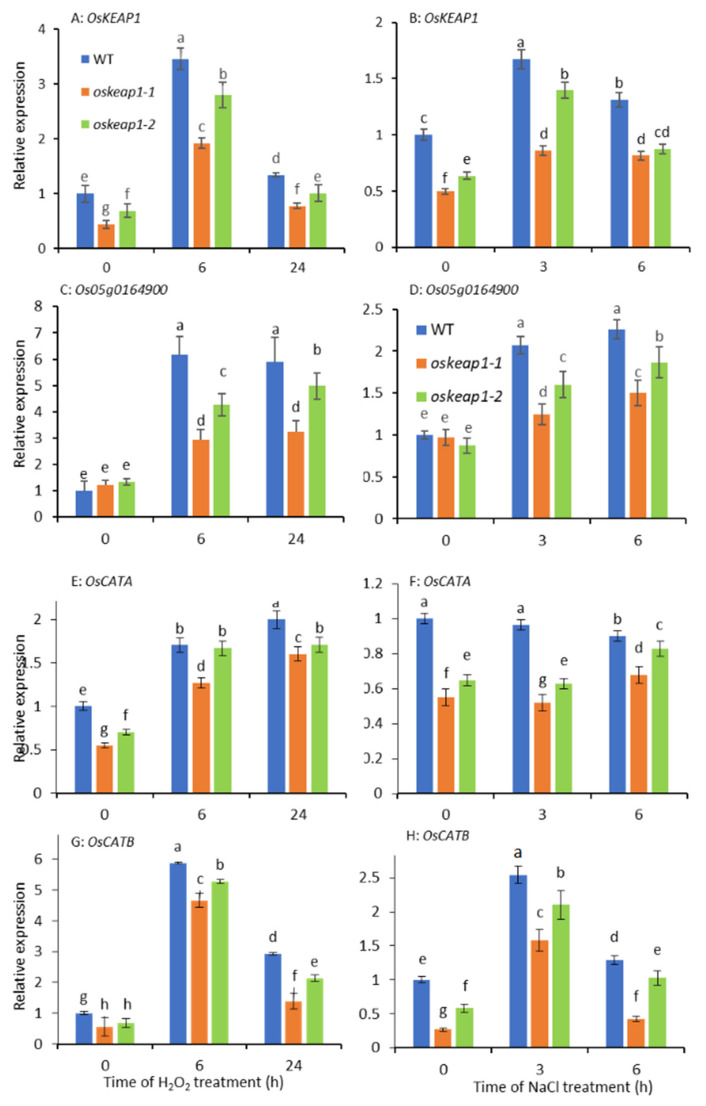
Relative expression of *OsKEAP1*, *Os05g0164900*, and two catalase genes (*OsCATA* and *OsCATB*) in leaf tissues of rice cultivar Xidao #1 (WT) and its two *oskeap1* mutants grown under normal conditions and subjected to salt (NaCl, 200 mM) and H_2_O_2_ (10 mM) treatment. *OsACTIN* was used as an internal reference and the expression levels were all compared with that of the WT grown under normal conditions (set as 1). Data are shown as mean ± standard error of 3 biological repeats. Data with different letters (a,b,c, etc.) represent significance at the 0.05 level.

To understand the biological mechanism leading to the redox changes in the *oskeap1* mutants, the expression of *OsKEAP1*, *Os05g0164900*, and two catalase genes was investigated in leaf tissue of plants grown under normal conditions and subjected to salt (NaCl, 200 mM) and H_2_O_2_ (10 mM) treatment (Figure 9).

The expression of *OsKEAP1* seemed to be responsive to both H_2_O_2_ and salt treatment, and the mRNA transcript levels of the two *oskeap1* mutants were significantly lower than those of the WT grown either under normal conditions or in media supplemented with H_2_O_2_ (Figure 9A) and salt (Figure 9B). A similar trend was observed for *Os05g0164900*, i.e., its expression was also responsive to these two stresses (Figure 9C,D). The *OsKEAP1* mutations did not affect the expression of *Os05g0164900* under normal conditions, but significantly lowered its expression under both stresses (Figure 9C,D).

To examine whether the *oskeap1* mutations affected the expression of genes involved in ROS degradation, the expression of two catalase genes, *OsCATA* and *OsCATB*, was investigated. The two genes seemed to have different responses to salt and H_2_O_2_ treatment. The former is responsive only to H_2_O_2_ treatment (Figure 9E,F), whereas the latter is responsive to both treatments (Figure 9G,H). The two *oskeap1* mutations seemed to have significantly repressed the expression of *OsCATA* and *OsCATB*, irrespective of treatment (Figure 9E–H).

## 3. Discussion

As a starting point to explore whether there is a ROS regulatory pathway in plants similar to the KEAP1-NRF2 pathway found in animals and other organisms, the present study first identified the most likely KEAP1 ortholog in rice (OsKEAP1), and then generated two knockdown mutants by targeted mutagenesis on its 5′ UTR. Further examination revealed that downregulation of *OsKEAP1* impaired plant growth and development, altered stress response and ROS accumulation in seedlings, and increased sensitivity to ABA in seed germination. This study not only identified and characterized the first probable KEAP1 ortholog in plants but also served as an example of a unique way of generating knockdown mutants by means of UTR editing.

The failure to generate knockout *oskeap1* mutants by us and other groups suggests that OsKEAP1 may play an indispensable role in rice growth and development and its complete knockout might be lethal. Based on this reasoning, we generated two downregulated mutants by targeting its 5′ UTR, which is known to regulate mRNA stability and translation [16]. Both *oskeap1* mutants had a 1-bp insertion in the same position of the 5′ UTR (Figure 3B). The former had a significantly greater negative effect than the latter (Figure 4). This seems to be consistent with their theoretical effect on the secondary structure of mRNA (Figure S4)—the A insertion in *oskeap1-1* is expected to result in the disappearance of the downstream loop, whereas the T insertion in *oskeap1-2* would only lead to an enlarged downstream loop. The different downregulation level of *OsKEAP1* in the two *oskeap1* mutants indeed enabled us to quantitatively link the function of OsKEAP1 to multiple phenotypes, as evidenced by the changes in almost all phenotypes, from agronomic traits and seed germination to responses to H_2_O_2_ and salt treatments, in proportion to the downregulation level in the two mutants.

OsKEAP1 was initially identified through in silico analysis in the present study. Because its amino acid identity to KEAP1 is relatively low (Figure 1A) and previous studies also did not identify a close KEAP1 ortholog in plants, the evidence of OsKEAP1 as the ortholog of KEAP1 in rice may not be sufficient. However, our study did prove that OsKEAP1 is involved in redox regulation, based on the following observations. First, OsKEAP1 has the key domain of KEAP1—the Kelch repeats domain (Figure 1C)—and the expression of *OsKEAP1* is responsive to H_2_O_2_ and salt treatment. Second, we demonstrated that the two mutants had significantly greater levels of H_2_O_2_, MDA, and proline than their wild-type Xidao #1 parent (Figure 8), suggesting that the downregulation of *OsKEAP1* impaired the ability to clean ROS in seedlings. Third, the expression of *OsCATA* and *OsCATB* was significantly downregulated in the two *oskeap1* mutants grown under normal conditions and under stresses (Figure 9C–F), suggesting that the ROS increase in the two mutants is related to the altered regulation of ROS response. However, the working mechanism of KEAP1-NRF2, if it exists in rice, seems already to be different from that found in animals. In humans and animals, the downregulation of KEAP1 would increase the expression of its partner, NRF2, and consequently activate the expression of downstream antioxidant genes [17]. In the present study, we observed the opposite trend—the H_2_O_2_ level was increased in the two mutants (Figure 8A), whereas the expression of two catalase genes was significantly decreased (Figure 9E–H). Further studies are needed to reveal the mechanism and explain how OsKEAP1 works differently from KEAP1 in ROS regulation. For this, the following observations may provide some clues: KEAP1 has a BTB-BACK domain, whereas OsKEAP1 has a DCD domain at the N terminus (Figure 1C), and OsKEAP1 is located in both the cytoplasm and nucleus (Figure 2), whereas animal KEAP1 is found only in the cytoplasm [18].

OsKEAP1 seems to have orthologs among different plant species, all of which have DCD domains and the Kelch repeats (Figures S2 and S3). DCD is a plant-specific domain involved in phytohormone response, plant development, and programmed cell death [19]. Indeed, a simple in silico search of NRF2 homologs in rice revealed OsABI5 [20], the rice ortholog of the *Arabidopsis* gene *ABA Insensitive 5* [21]) to be the top candidate, though it also has a lower amino acid identity (<30%) to NRF2. ABI5 is a basic leucine zipper transcription factor and plays a broad role in the regulation of seed germination and early seedling growth, as well as regulation of the response to abiotic stresses [22]. This finding is not only in line with the potential role of OsKEAP1 in phytohormone (abscisic acid, ABA) response, as shown by the DCD domain, it could also explain the increased sensitivity of the *oskeap1* mutants to ABA (Figure 7D) because downregulation of *OsKEAP1* is expected to upregulate *OsABI5*.

*OsKEAP1* is differently expressed in various plant organs and tissues (Figure S1), hence its mutational effect on different traits could be quite different. In addition to the anthers, *OsKEAP1* also is highly expressed in the inflorescence, pistil, and ovary. This might be the reason underlying the great decrease of seed-set in the two mutants, which could not be explained merely by pollen fertility reduction (Figure 6). We speculate that the viability of megaspores and other female organs also may have been negatively affected by the downregulation of *OsKEAP1* in the two mutants, although more experiments are needed to validate this.

Os05g0164900 is a close paralog of OsKEAP1. It has both DCD and Kelch-repeat domains (Figure 1C) and its expression is responsive to salt and H_2_O_2_ treatment (Figure 9C,D). Moreover, when subjected to salt and H_2_O_2_ treatment, its expression level was significantly lower in the two mutants than their WT parents, in a similar trend to that of *OsKEAP1* (Figure 9C,D). These features suggest that Os05g0164900 could be another potential KEAP1 ortholog in rice.

## 4. Materials and Methods

### 4.1. In Silico Identification and Characterization of KEAP1 Orthologs in Plants

The amino acid sequences of human KEAP1, downloaded from NCBI [23], were used as a query for a BLAST search in the NCBI database to search for the KEAP1 orthologs in rice. Multiple databases were further searched for the identification of *KEAP1* orthologous genes in rice, such as Gramene [24] and RAPDB [25]. The amino acid sequences of KEAP1 orthologs of other plant species such as maize and sorghum were downloaded from Gramene [24] or OrthoDB [26]. The conserved domains of KEAP1 were analyzed using the Unipro and SMART databases. The secondary structure of the UTR of *KEAP1* was predicted in UNAFold [27]. The tissue-specific expression profile of genes in rice was obtained from RiceXPro [28].

### 4.2. Construction of CRISPR/Cas9 Vector

The optimal base-pairing sequences of sgRNA targeting the 5′ UTR of *OsKEAP1* were identified using the CRISPR-P 2.0 tool (http://crispr.hzau.edu.cn/CRISPR2/). The following sgRNA target oligo was chosen and a *Bsa*I recognition site was added on both ends of KEAP1-F and its complementary sequence KEAP1-R and synthesized from Tsingke (Hangzhou, China). The two oligos were annealed using Annealing Buffer 5X (Beyotime) and ligated to linearized pHUN4c12 (digested by *Bsa*I, NEB). The recombinant vector (pHUN4c12-K5U) was transformed into *Escherichia coli* and the insertion of oligos was confirmed by sequencing (TSINGKE, Hangzhou, China). The pHUN4c12-K5U vector was then transformed into *Agrobacterium* (strain EHA105).

Healthy seeds of the *japonica* rice variety Xidao #1 were de-hulled and surface sterilized using 70% ethanol for 1 min and 1% NaClO for 30–40 min, followed by rinsing with sterile distilled water 4–5 times. These seeds were then cultivated on a 2N6 callus-inducing medium [29] for 1 month at 28 °C and a 16-h photoperiod. When the diameter of the callus reached ~4 mm, the calli were transformed with pHUN4c12-K5U via *Agrobacterium*-mediated transformation. The transformed calli were then grown on selective media supplemented with hygromycin and tetracycline according to the method of Liu et al. [30]. Surviving calli were grown into plantlets in regeneration media, and later transplanted in soil and grown into T_0_ plants.

### 4.3. Identification and Development of OsKEAP1 Mutants

The genomic DNA of T_0_ plants was extracted by the modified CTAB (cetyltrimethylammonium bromide) method [30] and the targeted region of *OsKEAP1* was PCR amplified using primer pair Pk1-F and Pk1-R, with the following program: 95 °C, 2 min, 35 cycles of a three-step reaction including 95 °C for 30 s, 55 °C for 30 s and 72 °C for 40 s, and a final extension at 72 °C for 8 min. The PCR products were examined using high resolution melting (HRM) analysis, according to the method of Li et al. [31], and putative mutant samples were then sequenced to detect mutations in the target region. Seeds from mutant T_0_ plants were harvested and grown into T_1_ plants and analyzed further for the confirmation of mutations in the UTR and absence of transfer DNA (T-DNA) according to the method of Basnet et al. [32].

### 4.4. Real-Time Quantitative-PCR and Subcellular Localization

For real-time quantitative PCR (qPCR) analysis, total RNA was extracted from above-ground tissues of 10-day-old rice seedlings using an RNAprep pure Plant Kit (Tiangen, Beijing, China) and reverse transcribed using a PrimerScript RT reagent kit with genomic DNA eraser (Takara, Dalian, China). A Hieff^TM^ qPCR SYBR^®^ Master Mix (Yeasen, Shanghai, China) was used for qPCR in a Roche Illuminator (Penzberg, Germany). The primer pair KPqpcr-F and KPqpcr-R was used for qPCR of *OsKEAP1*, qKP2-F, and qKP2-R for *Os05g0164900*, qCATA-F, and qCATA-R for *OsCATA*, and qCATB-F and qCATB-R for *OsCATB* (Table 1). The rice *Actin* gene was used as an internal reference. Three biological replicates were taken for each treatment and the relative expression levels were calculated using the 2^−^^△△^*^CT^* analysis method.

The complementary DNA (cDNA) of OsKEAP1 was obtained by PCR amplification using the forward primer KpGFP-F containing the EcoRI recognition site, and the reverse primer KpGFP-R containing the BamHI recognition site. The PCR product was cloned into the linear vector pTZM28-GFP (digested with EcoRI and BamHI) to produce a fusion gene with a green fluorescence protein (GFP) under the control of the cauliflower mosaic virus (CaMV) 35s promoter (named the *OsKEAP1::GFP* vector).

Rice protoplasts were isolated from 10-day-old rice seedlings. The *OsKEAP1::GFP* was co-transformed with a mCherry-labeled nucleus marker (*NLS-mCherry*) into the rice protoplasts using polyethylene glycol [33] and incubated for 12–16 h. The pCAMBIA 1301 GFP vector under the control of the CaMV 35S promoter was used as a control. The subcellular localization of fluorescent proteins in protoplasts was observed under an LSM780 fluorescence confocal microscope.

### 4.5. Agronomic and Seed Germination Tests of OsKEAP1 Mutants

Transgene-free homozygous mutant T_2_ lines were developed from transgene-free T_1_ plants and were tested in plots for their agronomic performance along with their wild-type parental line Xidao #1, at the Zhejiang rice breeding station in Hangzhou. The two mutant lines and their parent line Xiadao #1 were grown in a randomized design with three replicates. In each replicate, 48 (6 × 8) plants of each line were grown as a plot. Agronomic data, including tiller numbers, plant height, thousand-grain weight, seed setting rate, and other agronomic traits, were collected at the grain maturity stage [32]. Pollen fertility was studied by the iodine staining method [34] and observed under a microscope. Plot yield is the total grain yield of each replicate.

For regular germination tests, grains were soaked in water at 30 °C for 48 h and then transferred onto wet filter paper for germination at room temperature. The test was performed in triplicate, each with 300 grains. The number of germinated seeds was recorded daily for 6 days. For assessment of the effect of ABA on seed germination, grains were dehulled and normal-looking brown rice grains were selected for testing. The rice grains were surface sterilized by rinsing with 75% ethanol for 30–60 s, followed by soaking in 1% NaClO solution for 40 min. After rinsing in water 4–5 times, the sterilized grains were cultured on 1/2 MS medium [15] supplemented with 0 and 1 μM ABA. The test was performed in triplicate, each with 60 grains.

The sequence information for all PCR primers and DNA oligos is listed in Table 1.

### 4.6. Seedling Growth

For testing of the respective redox states and responses to stresses, healthy and uniform seeds of Xidao #1 and its two *oskeap1* mutants were soaked in distilled water for 48 h at 30 °C in an incubator and then germinated on wet filter paper in darkness for 48 h. Then the germinating seeds were grown in 1/2 MS liquid medium for 10 days in a growth chamber. The temperature was set at 24 °C/16 °C for day/night with 16 h of light, with an irradiance of 300 μmol m^−2^s^−1^ and relative humidity of 60–70%. Uniform 10-day-old seedlings were used for subsequent experiments. For salt stress treatment, NaCl was added into a liquid medium at a final concentration of 200 mM and grown for up to 6 h, and for oxidative treatment, H_2_O_2_ was added at the final level of 10 mM and grown-up for 24 h before seedlings were taken for measurement.

### 4.7. Measurement of H_2_O_2_, MDA and Proline Content

The H_2_O_2_ level was determined in seedlings without roots using an H_2_O_2_ detection kit (Solarbio, Shanghai, China), according to the manufacturer’s instructions, and the optical density was recorded at 415 nm. Samples (~0.1 g) were prepared in liquid nitrogen and immediately used for the determination of H_2_O_2_ content. Six biological replicates were performed for each treatment.

The content of MDA was determined according to the method of Jiang et al. [35]. About 0.1 g tissues were mixed with 10% trichloroacetic acid and centrifuged at 10,000× *g* for 20 min. The supernatant was blended with the same amount of thiobarbituric acid and incubated at 95 °C for 40 min. After being quickly cooled on ice, the mixture was centrifugated for 10 min at 12,000× *g*. The absorbances of the supernatant were determined at 450, 532, and 600 nm, respectively.

The content of proline was measured following the protocol published by He et al. [36]. About 0.1 g tissues were homogenized with 3% sulfosalicylic acid and centrifuged at 10,000× *g* for 20 min. The supernatant was mixed with an equal volume of acidic ninhydrin and glacial acetic acid and incubated in boiling water for 50 min. After being quickly cooled on ice, the homogenate was mixed with toluene, the aqueous phase was collected and their absorbance was measured at 520 nm. The proline content was calculated using a standard curve.

## 5. Conclusions

In this study, we identified a probable KEAP1 ortholog in rice (OsKEAP1) based on its amino acid identity and domain features and demonstrated its involvement in ROS accumulation and response. We demonstrated that the downregulation of *OsKEAP1* could significantly and negatively affect agronomic performance, including yield-related traits and increased sensitivity to ABA in seed germination. Based on elevated levels of H_2_O_2_, MDA, and proline and decreased expression of two catalase genes in the *oskeap1* mutant seedlings, it was deduced that OsKEAP1 is involved in ROS regulation in rice, but in a way that is different from that of KEAP1. More studies are worthwhile to reveal the roles and working mechanisms of OsKEAP1 in rice growth, development, and responses to various internal and external stresses.

## Figures and Tables

**Table 1:** List of primers used in this study.

Name of Primers	Sequences (5′-3′)	Remarks
KEAP1-F	ggcaGGGTGCTACGAAACCCGC	*OsKEAP1* sgRNA synthesize
KEAP1-R	aaacGCGGGTTTCGTAGCACCC	
Pk1-F	GGTTGATCGATGCTTGCTGC	For *oskeap1* mutant identification and high resolution melting (HRM) analysis
Pk1-R	CACCAATCGCGACCAAATCG	
KPqpcr-F	CAAGCACTGGCCAGCTTAAT	q-PCR analysis
KPqpcr-R	GATTAGCGCGAACAGGAGCA	
qKp2-F	GCAGCCCGTCTATGATGAACTCTC	
qKp2-R	AGAAACCCTCCCTTGGGTCATAC	
qCATA-F	TCCCAGTGTGATGAGTCGTTGG	
qCATA-R	TCTTCACATGCTTGGCTTCACG	
qCATB-F	TCCTACTGGTCGCAGTGTGATG	
qCATB-R	TTTCAGGTTGAGACGTGAAGCC	
KpGFP F	gaattcATGGGTGCTGGAAAGAAGACTCA	Complementary DNA amplification
KpGFP R	ggatccCAATGGCAACGGCGCATGC	
HYG-F	AGAAGAAGATGTTGGCGACCT	Hygromycin marker primer
HYG-R	GTCCTGCGGGTAAATAGCT	

## Supplementary Materials

The following are available online at https://www.mdpi.com/2223-7747/9/11/1450/s1, Figure S1: Relative expression of OsKEAP1 in different rice tissues, Figure S2: Molecular Phylogenetic analysis of KEAP1 orthologs in plants, Figure S3: Domain analysis of KEAP1 ortholog in 10 plant species, Figure S4: Prediction of secondary RNA structure of two OsKEAP1 mutants, each with a single nucleotide insertion in its untranslated regions.
