# Supplementary material for: Identification, Characterization, and Mutational Analysis of a Probable KEAP1 Ortholog in Rice (Oryza sativa L.)
Source: Plants (Basel). 2020 Oct 27;9(11):1450. doi: 10.3390/plants9111450 (PMC7716215; doi:10.3390/plants9111450)
Supplement: Supplementary file 1 [file plants-09-01450-s001.zip › plants-896087-supplementary.docx]

**Supplementary Information**

**Identification, Characterization and Mutational Analysis of a KEAP1 Orthologue in Rice (*Oryza sativa* L.)**

**Yan-Hua Liu^1^, Meng Jiang^1^, Rui-Qing Li^2^, Rasbin Basnet^1^, Jian-Zhong Huang^1,3^, Shi-Yong Song^1^ and Qing-Yao Shu^1,^***

^1^ National Key Laboratory of Rice Biology and Zhejiang Key Laboratory of Crop Germplasm Resources, College of Agriculture and Biotechnology, Institute of Crop Sciences, Zhejiang University, Hangzhou, 310058, China

^2^ College of Agronomy, Anhui Agricultural University, Hefei 230036, China

^3^ Institute of Nuclear Agricultural Sciences, Key Laboratory for Nuclear Agricultural Sciences of Zhejiang Province and Ministry of Agriculture and Rural Affairs, Zhejiang University, Zijingang campus, 310058, China

***** Correspondence: Corresponding Author (e-mail: [qyshu@zju.edu.cn](mailto:k.h.engel@wzw.tum.de))


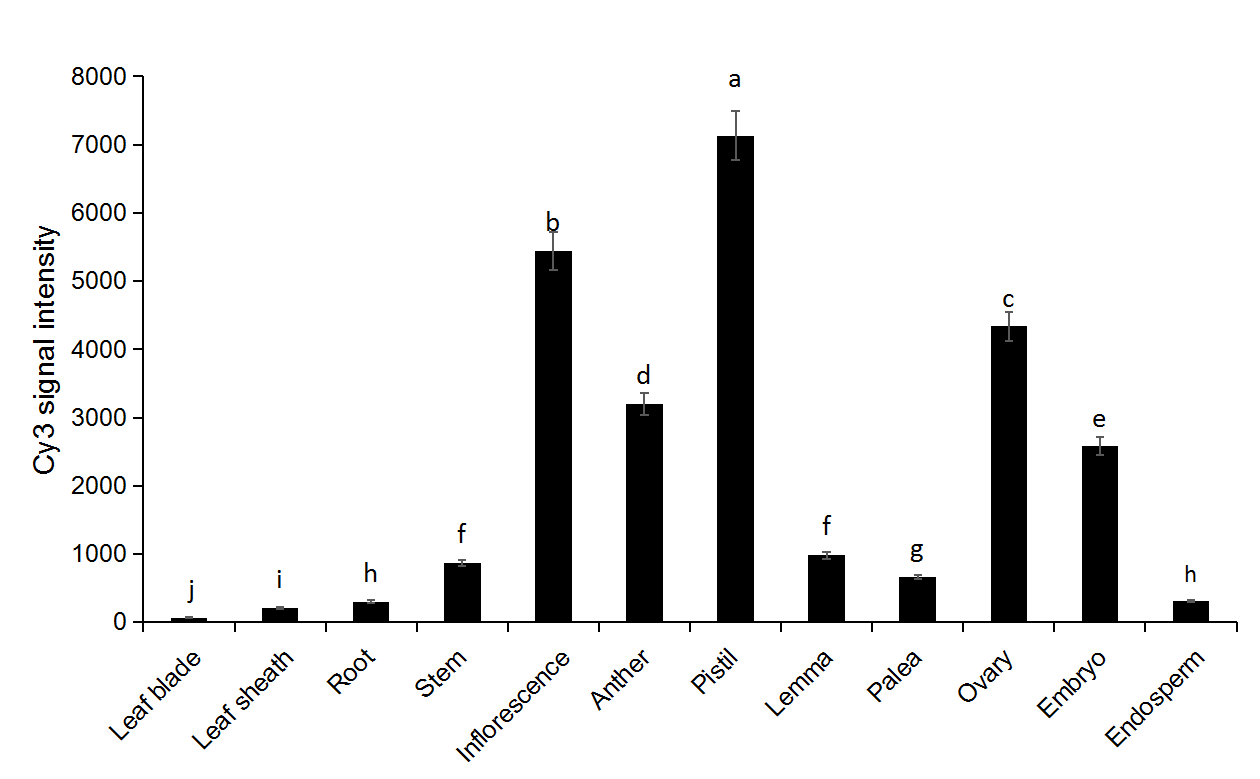


**Figure S1.**  Relative expression of *OsKEAP1* in different rice tissues. All values represent mean ± standard deviations of three replications. Student’s *t*-test, different letters represent significance at 0.05 level. Source: RiceXPro (https://ricexpro.dna.affrc.go.jp/）


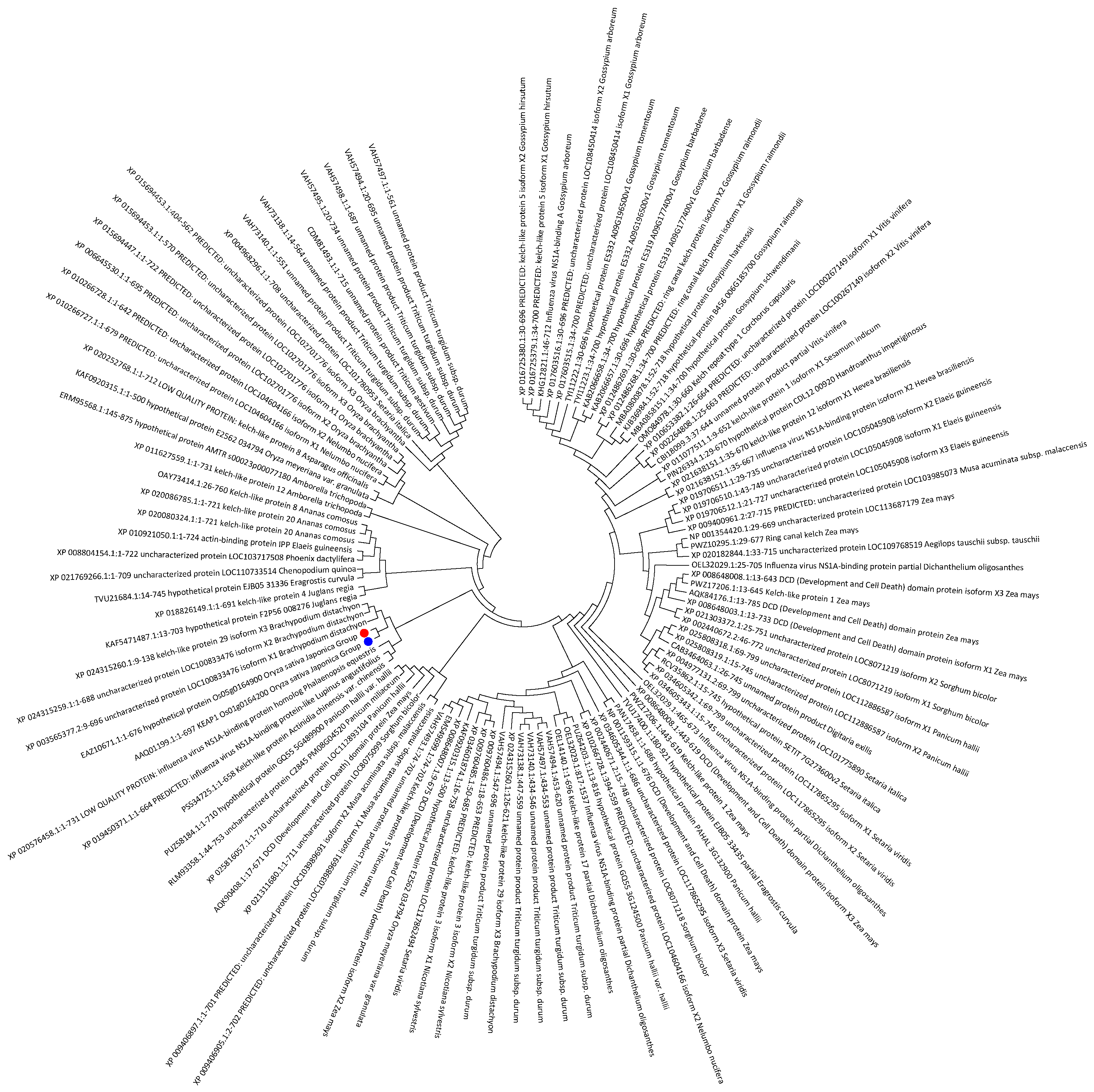


**Figure S2. Molecular Phylogenetic analysis of KEAP1 orthologs in plants.** The evolutionary history was inferred by using the Maximum Likelihood method based on the JTT matrix-based model. The tree is drawn to scale, with branch lengths measured in the number of substitutions per site. The analysis involved 110 amino acid sequences, with OsKAEP1 and . All positions containing gaps and missing data were eliminated. Evolutionary analyses were conducted in MEGA7.


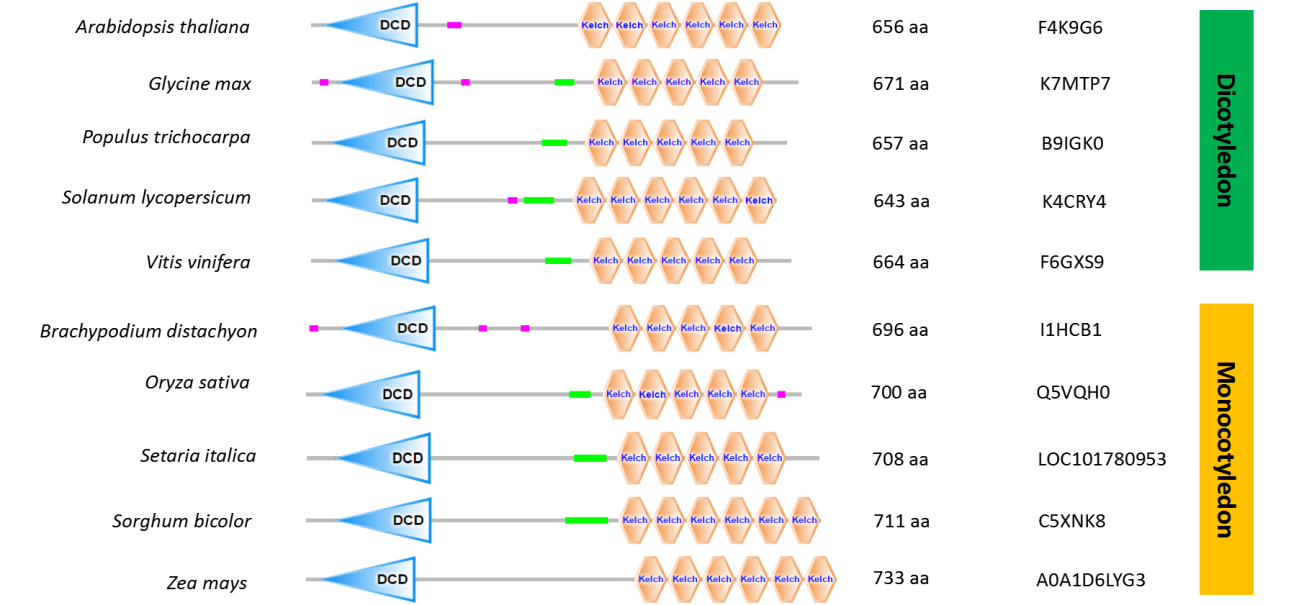


**Figure S3.**  Domain analysis of of KEAP1 ortholog in 10 plant species.


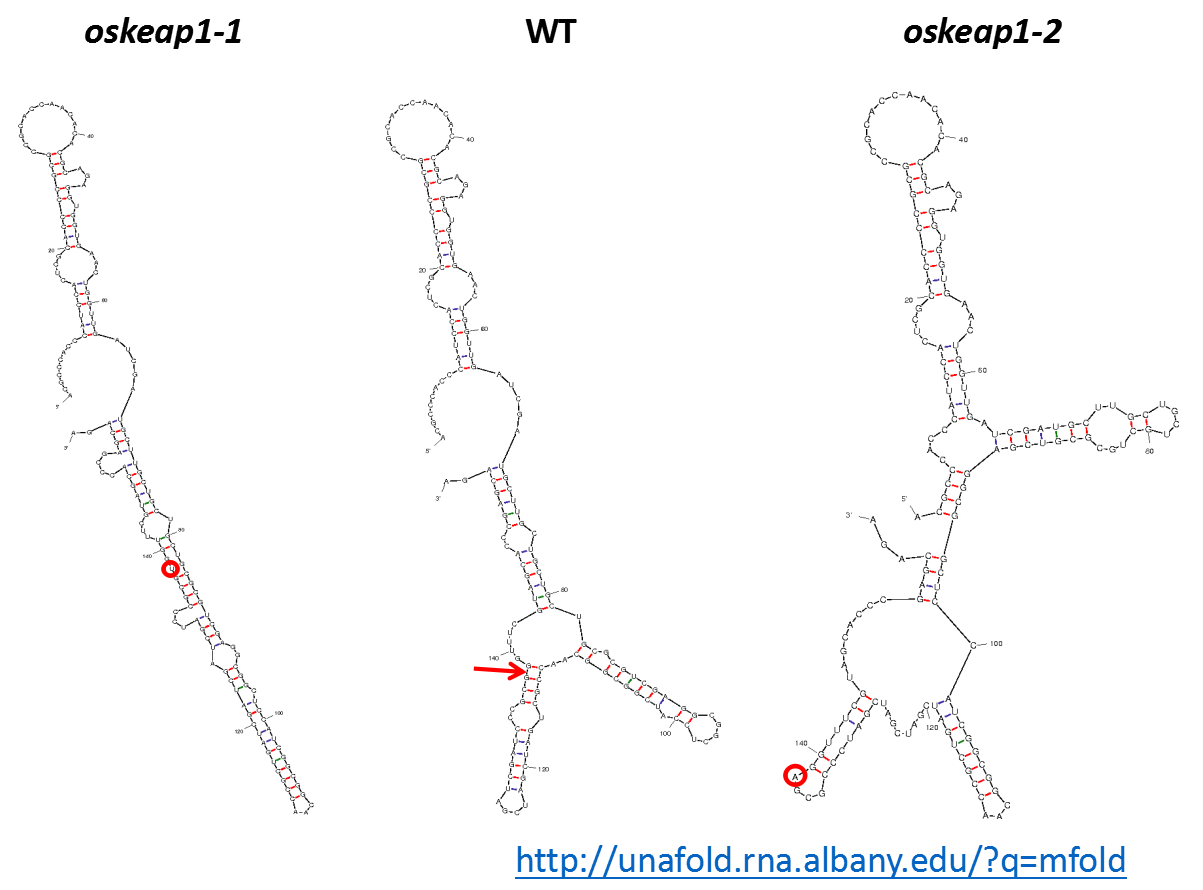


**Figure S4.**  Prediction of secondary RNA structure of two *OsKEAP1* mutants, each with a single nucleotide insertion in its untranslated regions
